# Unveiling a classical mutant in the context of the GH3 β-glucosidase family in *Neurospora crassa*

**DOI:** 10.1186/s13568-023-01658-0

**Published:** 2024-01-05

**Authors:** Yuxin Zhang, Basant Nada, Scott E. Baker, James E. Evans, Chaoguang Tian, J. Philipp Benz, Elisabeth Tamayo

**Affiliations:** 1 Fungal Biotechnology in Wood Science, Holzforschung München, TUM School of Life Sciences, Technical University of Munich, 85354 Freising, Germany; 2 DOE Joint BioEnergy Institute, Emeryville, CA 94608 USA; 3 Microbial Molecular Phenotyping Group, Environmental Molecular Sciences Division, Pacific Northwest National Laboratory, Richland, WA 99354 USA; 4 Key Laboratory of Engineering Biology for Low-Carbon Manufacturing, Tianjin Institute of Industrial Biotechnology, Chinese Academy of Sciences, Tianjin, 300308 China; 5 National Technology Innovation Center of Synthetic Biology, Tianjin, 300308 China

**Keywords:** β-glucosidase, *gluc-1* mutant, *Neurospora crassa*, Cellobiose utilization

## Abstract

Classical fungal mutant strains obtained by mutagenesis have helped to elucidate fundamental metabolic pathways in the past. In the filamentous fungus *Neurospora crassa*, the *gluc-1* strain was isolated long ago and characterized by its low level of β-glucosidase activity, which is essential for the degradation of cellulose, the most abundant biopolymer on Earth and the main polymeric component of the plant cell wall. Based on genomic resequencing, we hypothesized that the causative mutation resides in the β-glucosidase gene *gh3-3* (*bgl6, NCU08755*). In this work, growth patterns, enzymatic activities and sugar utilization rates were analyzed in several mutant and overexpression strains related to *gluc-1* and *gh3-3*. In addition, different mutants affected in the degradation and transport of cellobiose were analyzed. While overexpression of *gh3-3* led to the recovery of β-glucosidase activity in the *gluc-1* mutant, as well as normal utilization of cellobiose, the full gene deletion strain Δ*gh3-3* was found to behave differently than *gluc-1* with lower secreted β-glucosidase activity, indicating a dominant role of the amino acid substitution in the point mutated *gh3-3* gene of *gluc-1*. Our results furthermore confirm that GH3-3 is the major extracellular β-glucosidase in *N. crassa* and demonstrate that the two cellodextrin transporters CDT-1 and CDT-2 are essential for growth on cellobiose when the three main *N. crassa* β-glucosidases are absent. Overall, these findings provide valuable insight into the mechanisms of cellulose utilization in filamentous fungi, being an essential step in the efficient production of biorefinable sugars from agricultural and forestry plant biomass.

**Supplementary Information:**

The online version contains supplementary material available at 10.1186/s13568-023-01658-0.

## Introduction

Cellulose degradation is a key step in the production of biorefined products from agricultural and forestry plant biomass. Its hydrolysis requires the action of three main enzymatic activities: endoglucanases (EC 3.2.1.4), which randomly cleave internal bonds of the cellulose polymer, exoglucanases (EC 3.2.1.91) which attack the ends of the cellulose chain to produce cellobiose, and β-glucosidases (EC 3.2.1.21), responsible for converting cellobiose into glucose (Horn et al. 2021).

The model filamentous fungus *Neurospora crassa* has been used for decades to study the mechanisms and regulation of enzymes that degrade cellulose (Eberhart et al. 1977; Yazdi et al. 1990; Romero et al. 1999; Tian et al. 2009; Schmoll et al. 2012; Znameroski et al. 2012; Coradetti et al. 2013; Gabriel et al. 2021). In *N. crassa*, the beta-glucosidase system has been well characterized for sugar utilization-related biotechnological applications (Eberhart et al. 1964; Galazka et al. 2010; Karkehabadi et al. 2018). This fungus contains seven β-glucosidase genes in its genome (*gh1-1*, and *gh3-1* to *gh3-6*) belonging to glycoside hydrolase (GH) families 1 and 3 (Galagan et al. 2003; Wu et al. 2013a). Three of them, GH1-1 (NCU00130), GH3-4 (NCU04952) and GH3-3 (NCU08755), are the major contributors of β-glucosidase activity in this fungus, with GH1-1 having an intracellular location and GH3-3 and GH3-4 extracellular location, with the majority of the GH3-4 activity found in the supernatant and most of the GH3-3 activity bound to the cell wall (Wu et al. 2013b). Deletion of their encoding genes resulted in the loss of most of the overall β-glucosidase activity (Znameroski et al. 2012). Wu et al. (2013b) came to the same conclusion after studying the β-glucosidase activity in sextuple mutants, in which only one of the seven genes was expressed. In their study, and after induction with Avicel, only sextuple deletion strains with one of the three mentioned β-glucosidase genes remaining intact showed β-glucosidase activity different from that of the septuple deletion strain, which had all seven β-glucosidase genes deleted and displayed no β-glucosidase activity in any cellular location. In addition to cellulose deconstructing enzymes, cellodextrin transporters are also involved in the process of cellulose degradation and utilization (Cai et al. 2014; Li et al. 2019; Havukainen et al. 2020; Lenz et al. 2022). In *N. crassa*, the cellodextrin transporters CDT-1 and CDT-2 contribute to cellulose sensing and the ∆*cdt-2* mutant was previously observed to be severely impaired for growth on cellulose (Galazka et al. 2010; Znameroski et al. 2014).

Thanks to the generation and wide dissemination of the *N. crassa* gene knockout library (Dunlap et al. 2007), many of the enzymes and regulatory pathways involved in *N. crassa* biomass degradation have been analyzed to date (Znameroski and Glass 2013; Seibert et al. 2016; Huberman et al. 2016). In many cases however, connections to previous literature and classical genetic mutant strains are missing. Eberhart et al. (1964) performed a pioneer study of the β-glucosidase system of *N. crassa* consisting of a screening for *N. crassa* β-glucosidase loss-of-function strains after irradiation of a wild-type strain with ultraviolet light. The *gluc-1* mutant strain was isolated having consistently lower β-glucosidase activity than the wild-type strain (Eberhart et al. 1964) and it was further utilized for the study of the system (Eberhart and Beck 1970; Perkins et al. 1982). It was found that only thermostable β-glucosidase activity was significantly reduced in this mutant (~ 90%), whereas thermolabile glucosidase was unaffected (Mahadevan and Eberhart 1964; Eberhart and Beck 1970). McCluskey and Baker initiated a genome re-sequencing project, allowing for the putative identification of the mutations associated with corresponding classical mutant phenotypes, including that for *gluc-1* (McCluskey et al. 2011; McCluskey and Baker 2022). However, experimental evidence of the mutation being causative for the observed phenotype is lacking.

In this study, we aimed to verify the proposed link between *gh3-3* and *gluc-1*. To this end, relevant mutant strains of *N. crassa* were assayed and the β-glucosidase GH3-3 expressed in the *gluc-1* mutant background, to see if the phenotype could be rescued. Moreover, other mutants affected in cellobiose uptake and degradation were analyzed for an improved mechanistic understanding of the underlying utilization system.

## Materials and methods

### Biological material and growth conditions

*N. crassa* strains used in this study are listed in Table 1. *N. crassa* strains were grown on 2% sucrose Vogel’s minimal medium slants in the dark at 30 °C for 2 days, and transferred to light/dark cycle conditions at 25 °C for conidiation. Histidine was added to the medium when required.

**Table 1:** *Neurospora crassa* strains used in this work

Strain	Relevant genotype	References
WT	Oak Ridge wild-type	FGSC#2489
*gluc-1*	FGSC#1224, *gh3-3* mutant, mat a	Eberhart et al. (1964); McCluskey and Baker (2022)
∆*gh3-3*	Δ*NCU08755*::*hph*, mat a	FGSC#18387
WT *his-3*	WT *his-3*, mat A	FGSC#6103
*gluc-1 his-3*	FGSC#1224 *his-3* (Crossing of FGSC#6103 and FGSC#1224)	This work
*gluc-1* p*N::gh3-3*	*gluc-1 his-3* transformed with p*N*::*gh3-3*	This work
*gluc-1* p*ccg-1::gh3-3*	*gluc-1 his-3* transformed with p*ccg-1::gh3-3*	This work
F6	Sextuple *bgl* deletion strain (*gh3-3* active)	Wu et al. (2013a)
∆3βG	∆*gh1-1*∆*gh3-3*∆*gh3-4*	Cai et al. (2015)
∆3βG∆*cdt-1*∆*cdt-2*	∆*gh1-1*∆*gh3-3*∆*gh3-4*∆*cdt-1*∆*cdt-2*	Cai et al. (2015)
∆7βG	FGSC#2489, no active β-glucosidase genes	Wu et al. (2013b)

### Nucleic acids extraction

Genomic *N. crassa* DNA was extracted from conidia using phenol:chloroform:isoamylalcohol (25:24:1) separation and ethanol precipitation. 30–100 mg of frozen harvested conidia were homogenized using a bead beater (1 min at maximum speed) after adding lysis buffer (50 mM NaOH, 1 mM EDTA, 1% (v/v) Triton X-100). Extracted gDNA was stored at -20° C until used.

### Gene isolation and constructs

For constitutive expression of *gh3-3*, the full-length genomic DNA of *gh3-3* was obtained by PCR amplification of wild-type *N. crassa* genomic DNA using the primers ‘p*ccg Xba*I *gh3-3* FW’ and ‘p*ccg Pac*I *gh3-3* RV’. Restriction digestion with the enzymes *Xba*I and *Pac*I was carried out on the fragments and the pCCG backbone plasmid (Honda and Selker 2009), containing 5′ and 3′ *his-3* homology regions and the constitutive promoter of *ccg-1*, and fragments were ligated with the T4 DNA ligase (NEB, Ipswich, MA, USA). For the expression under control of the native promoter of *gh3-3*, the primers ‘native *gh3-3’* and ‘native *gh3-3* RV part2’ were used for *gh3-3* amplification, to include the 1000 bp upstream of the start codon. All primers used are listed in Table 2. Successfully amplified fragments were isolated using the HiYield PCR Clean-up/Gel Extraction Kit (SLG^®^, Gauting, Germany) and assembled via Gibson assembly (Gibson et al. 2009) using the NEBuilder^®^ HiFi DNA Assembly cloning kit (NEB, Ipswich, MA, USA), followed by transformation of *Escherichia coli* TG-1 (Zymo Research Europe GmbH, Freiburg, Germany) following standard procedures and purified using the Hi Yield® Plasmid Mini DNA Isolation Kit (SLG^®^, Gauting, Germany). All plasmids were checked by sequencing using an automated DNA sequencer (Eurofins Genomics, Germany GmbH, Ebersberg, Germany) before further use.

**Table 2:** Oligonucleotides used in this study

Primer	Sequence 5′–3′	Application
p*ccg Xba*I *gh3-3* FW	aaaaTCTAGAATGAAGTTCGCCATTCCGCT	Cloning in p*ccg-1* plasmid
p*ccg Pac*I *gh3-3* RV	aaaaTTAATTAATCAGGGAAGAACCTCCTCGAG	Cloning in p*ccg-1* plasmid
*gh3-3* native FW part2	TCTCGAGGAGGTTCTTCCCTGAggcggaggcttaatcggctt	Cloning in plasmid with native promoter (p*N*)
AQUA_AmpR_ColE1_pYTK095_RV	acggttatccacagaatcaggg	Cloning in p*N* plasmid
AQUA_cPCR2_fw	ctgcgttatcccctgattctgtg	Cloning in p*N* plasmid
native *gh3-3* RV part1	GGCCCAATGGGACTTGGCATCCTgatggactgctccttctagcg	Cloning in p*N* plasmid
native *gh3-3*	AGGATGCCAAGTCCCATTGGGCC	Cloning in p*N* plasmid
native *gh3-3* RV part2	TCAGGGAAGAACCTCCTCGAG	Cloning in p*N* plasmid
p*ccg* native *gh3-3* sequencing primer 1	CCGATGCCGAACATCTGTTCA	Sequencing of *gluc-1*
Tail-hph3	CGACAGACGTCGCGGTGAGTTCAG	Genotyping
*gh3-3* FW	CTGTTACGGCGGATATCACCAACACG	Genotyping
*gh3-3* RV	AAACGAGCCCAGTTGACTCCACATGC	Genotyping
*gh3-3* genotyping checking primer FW	GGGATGTGAGTTCATGGGATACGG	Genotyping
p492_his3_check_R	GTCAGCATCCGTCTTGAGCAC	Genotyping

### Expression in *N. crassa* and growth assays

For homologous expression analyses, the *N. crassa gluc-1 his-3* strain was obtained by crossing the WT *his-3* strain with the *gluc-1* strain (Table 1) and transformed with the finished constructs by electroporation.

For the plate assays, conidia of different *N. crassa* strains were harvested, washed and plated on VMM + 1% sugar (sucrose or cellobiose) + 0.11 M Tris (pH 8) + 1.5% agar and incubated at 25 °C for 7 days. Tris was added to the medium to avoid overgrowth of the strain on the plate and to have a colony shape (Huberman et al. 2017). For the liquid growth assays, strains were grown in 24-well plates which were directly inoculated with 10^6^ conidia/ml in a volume of 3 ml medium. Cultures were grown in 1% sucrose or 1% cellobiose for 3 days and in 1% carboxymethylcellulose (CMC, Sigma, St. Louis, USA) for 8 days at 25 °C, 200 rpm and constant light. To determine the biomass of the strains, the mycelial mass was dried for 16 h on aluminum pans at 105 °C and measured afterwards. All assays were performed with biological triplicates for each strain per each condition.

### Enzymatic assays

*N. crassa* conidia were inoculated into 3 ml of Vogel’s medium containing 2% sucrose at a final concentration of 10^6^ conidia/ml and cultured at 25 °C, 200 rpm and constant light for 20 h. The resulting mycelia were washed in carbon-free Vogel’s medium three times, transferred to Vogel’s medium with 1% Avicel as the sole carbon source for induction and cultured at 25 °C, 200 rpm and constant light for 16 and 48 h. Supernatants containing the secreted enzymes were taken, aliquoted and stored at − 80 °C until use. The β-glucosidase activity was measured as described by Wu et al. (2013b) with some modifications. To inactivate the thermolabile β-glucosidases, the supernatants were incubated at 60 °C for 1 min. As a preparatory step, a reaction mixture of 50 μl of 100 mM NaAc (pH 5.0) and 25 μl of 20 mM p-nitrophenyl-β-D-glucopyranoside was incubated on ice for 5 min. For the β-glucosidase activity assay, a volume of 25 μl of each resulting supernatant sample was added to the reaction mixture and incubated at 37 °C for 20 min. The reaction was quenched by adding 100 μl of 200 mM Na_2_CO_3_ solution. For blank wells, supernatants were added after quenching. The absorbance of the released p-nitrophenol was measured at 400 nm. Cellulase activity was measured using AZO-CM-Cellulose (Megazyme, Wicklow, Ireland), according to manufacturer’s instructions. All assays were done with biological triplicates for each strain per each condition.

### Cellobiose utilization

*N. crassa* conidia were inoculated into 3 ml of Vogel’s medium containing 10 g/l cellobiose as the sole carbon source at a final concentration of 10^6^ conidia/ml in 24-well plates. Plates were cultured at 200 rpm at 25 °C and constant light. Samples of 50 µl were taken at different times. The amount of total reducing sugars in the supernatant (glucose + cellobiose) was determined using the dinitrosalicylic acid (DNSA) assay (Miller 1959; Gonҫalves et al. 2010).

### Statistical analyses

Data shown in Figs. 3–4 represent the mean of at least three biological replicates and error bars correspond to the standard error. For each parameter analyzed, each treatment was first subjected to the Shapiro–Wilk test for normality. If treatments had a normal distribution, a two-tailed Student’s T-test was performed. In case one (or both) of the treatments were not normally distributed, a Mann–Whitney U test was applied. Significance is indicated by asterisks (*p < 0.05; **p < 0.01; ***p < 0.001). For data comparing more than two groups (Fig. 5), when showing a normal distribution, an ANOVA and Tuckey post hoc test were applied. Significant differences with p < 0.05 are indicated by different letters.

### Phylogenetic analysis

The amino acid sequences of 111 orthologues of *N. crassa* GH3-3 from 10 biotechnologically relevant fungi were downloaded from FungiDB (fungidb.org) and manually curated. Full-length of amino acid sequences were aligned by Clustal Omega (Sievers and Higgins 2018; https://www.ebi.ac.uk/Tools/msa/clustalo/). Alignments were imported into the Molecular Evolutionary Genetics Analysis (MEGA) package version 11 (Tamura et al. 2021). A phylogenetic analysis was conducted by the neighbour-joining (NJ) method, implemented in MEGA, with a pair-wise deletion of gaps and the Poisson model for distance calculation. Bootstrap analyses were carried out with 1000 replicates. The evolutionary tree was drawn to scale.

### Protein structural analysis

The three-dimensional structural model of GH3-3 in its mutated (*gluc-1*) form was produced from the Swiss-Model server and PDB: 5nbs.1.A was used as a template for the Gluc-1 (Waterhouse et al. 2018; Guex et al. 2009). The structural alignment between Gluc-1 model and GH3-3 (PDB: 5nbs.1.A) was visualized in PyMOL software (Schrödinger and DeLano 2020).

## Results

### Identification of the *gluc-1* mutation

**Fig. 1:**
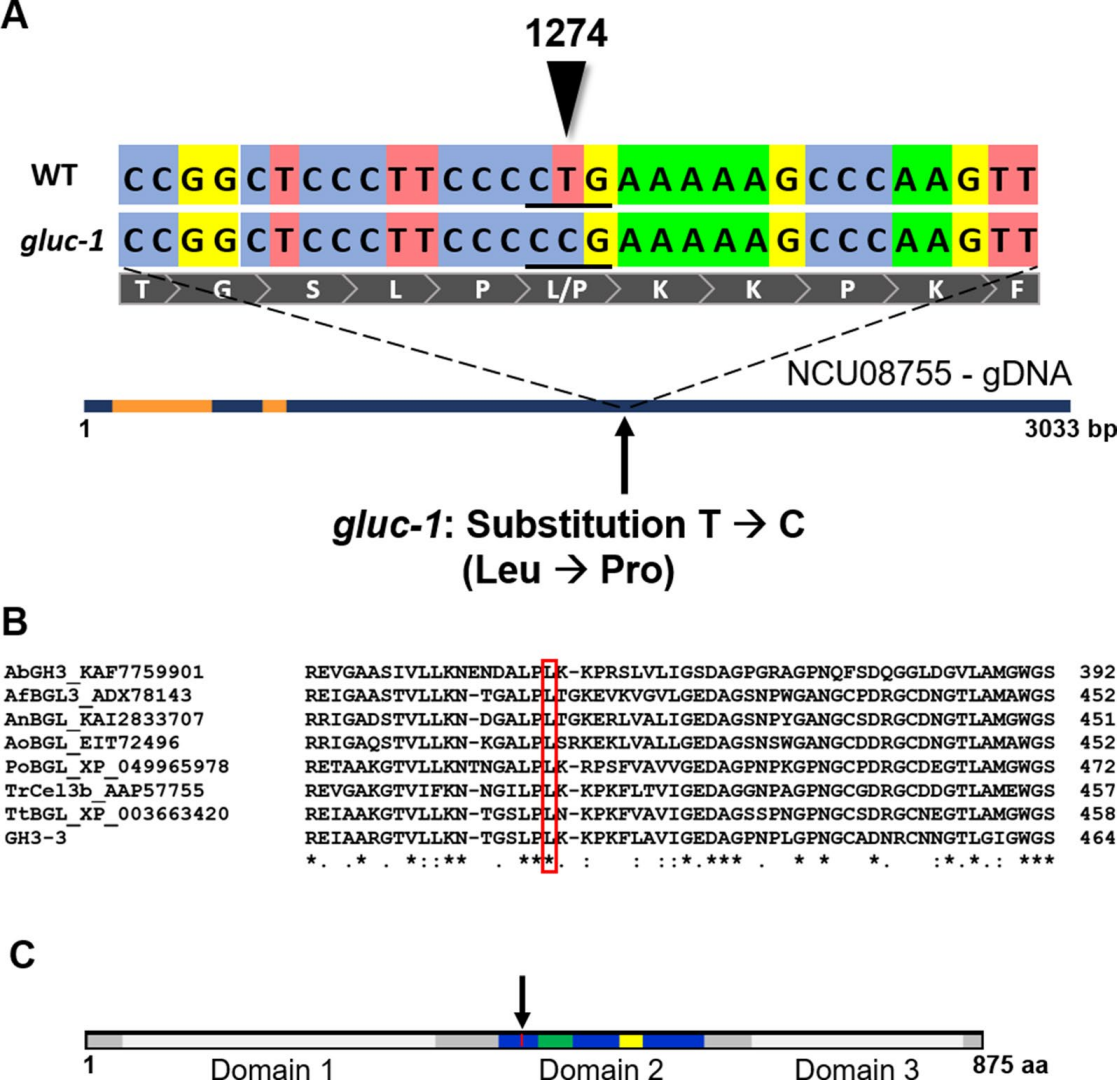
Schematic of the mutation in the *gluc-1* mutant and conservation analysis. **A**. Sequencing analysis revealed that *gluc-1* mutant has a mutation in NCU08755, a member of the beta-glucosidase family 3 of glycoside hydrolases. The *gluc-1* mutation at nucleotide residue 1274 leads to a substitution of leucine for proline at amino acid residue 425. The nucleotide area surrounding the *gluc-1* mutation is shown for the wild-type (WT, top) and the mutant (bottom) alleles. The encoded amino acid sequence is shown below in grey. The genomic DNA is represented below, with introns in orange. **B**. Alignment of GH3-3 and other closely related β-glucosidases of various filamentous fungi. The conserved residue 425 is indicated by a red box. Organisms: Ab *Agaricus bisporus*, Af *Aspergillus fumigatus*, An *Aspergillus niger*, Ao *Aspergillus oryzae*, Po *Penicillium oxalicum*, Tr *Trichoderma reesei*, Tt, *Thermothelomyces thermophilus*. **C**. Schematic of domains in the GH3-3 amino acid sequence (acc. to Karkehabadi et al. 2018). Domains 1 and 3 are depicted in light grey. The α/β region of domain 2 is colored in blue. Loops III and IV are colored in green and yellow, respectively. Amino acid residue 425 is shown in red and indicated by an arrow

Recently, the genome of the *N. crassa* classical mutant strain *gluc-1* (FGSC 1224) was re-sequenced, and the underlying mutation hypothesized to reside in the gene *NCU08755* (*gh3-3*), encoding for the β-glucosidase GH3-3 (McCluskey and Baker 2022). GH3-3 is a member of the GH3 family with extracellular localization (Wu et al. 2013b). The *gh3-3* gene was found to contain a substitution of one thymine by a cytosine, resulting in a change of leucine to proline at amino acid residue 425 (Fig. 1A), a residue that is conserved in fungal protein orthologues (Fig. 1B; Xia et al. 2016; Mohsin et al. 2019) and located in domain 2 (Fig. 1C; Karkehabadi et al. 2018). This domain was shown to be an α/β-domain and contains two loops that constitute one side of the active site cleft (Karkehabadi et al. 2018).

### Phylogenetic analysis of *N. crassa* β-glucosidases

**Fig. 2:**
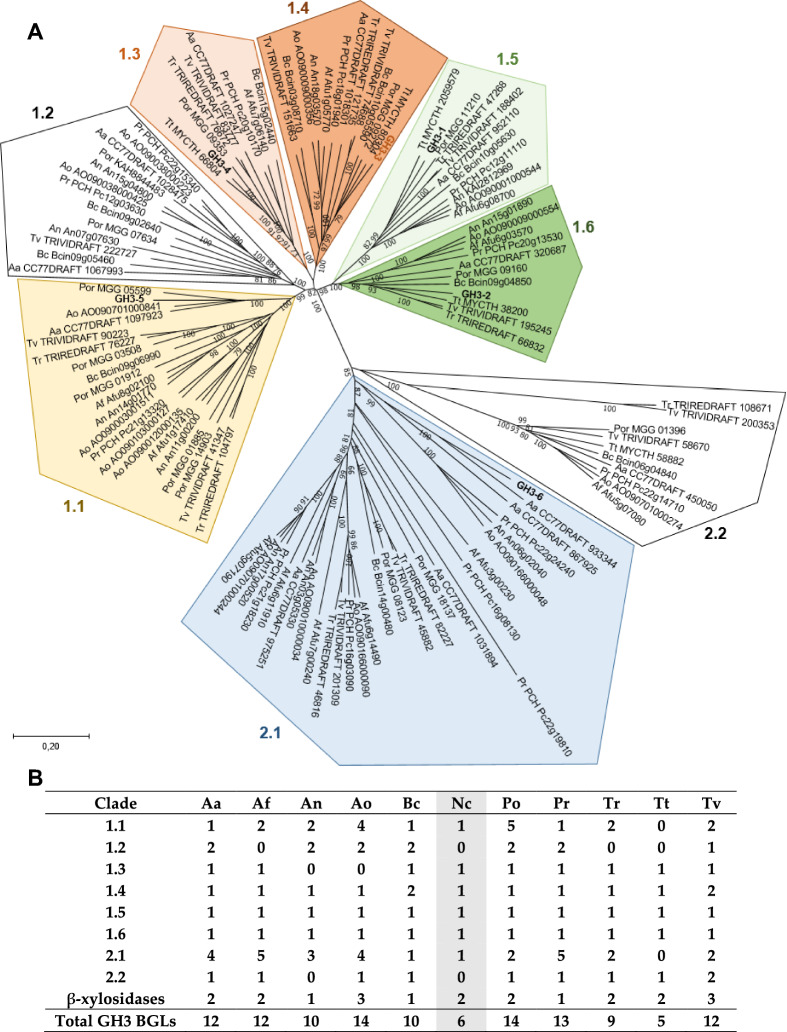
**A**. Phylogenetic analysis of GH3 family protein with β-glucosidases from various filamentous fungi. The unrooted neighbor-joining tree was created with MEGA11. NCBI accession numbers are indicated. *N. crassa* GH3-3 is shown in brown bold. The other *N. crassa* β-glucosidases are shown in bold. Related clades are in similar colors. Clades without any β-glucosidase from *N. crassa* are left white. Bootstrap values above 70 and supporting a node used to define a cluster are indicated. **B**. Number and phylogeny of the β-glucosidase and β-xylosidase orthologues belonging to the GH3 family identified in the genome of different fungi. Organisms: Aa *Alternaria alternata*, Af *Aspergillus fumigatus*, An *Aspergillus niger*, Ao *Aspergillus oryzae*, Bc *Botrytis cinerea*, Nc *Neurospora crassa*; Po *Pyricularia oryzae*, Pr *Penicillium rubens*, Tr *Trichoderma reesei*, Tt *Thermothelomyces thermophilus*, Tv *Trichoderma virens*

In order to study the phylogenetic relationships of the β-glucosidases of *N. crassa*, focusing mainly on GH3-3, we used GH3-3 to search for orthologues from different fungi in FungiDB (fungidb.org). The search retrieved not only β-glucosidases of the GH3 family but also β-xylosidases belonging to the same family (*N. crassa* possesses two β-xylosidases belonging to this family in its genome: GH3-7 (NCU09923) and GH3-8 (NCU00709); Wang and Arioka 2021). The phylogenetic analysis (Fig. 2) showed that GH3-3 has high homology to its homologs in other fungi (clade 1.4), as previously observed by Znameroski et al. (2012). Overall, eight clades were identified, with two being more distantly related to the rest (Fig. 2A; clades 2.1 and 2.2). The six *N. crassa* β-glucosidases of family GH3 clustered in clades 1.1 (GH3-5), 1.3 (GH3-4), 1.4 (GH3-3), 1.5 (GH3-1), 1.6 (GH3-2), and 2.1 (GH3-6). Close homology was found for GH3-1 and GH3-2 as well as for GH3-3 and GH3-4, respectively, which are located in highly related neighboring clades. GH3-6 is the most distantly related to the rest, belonging to clade 2.1. Clades 1.2 and 2.2 do not contain any β-glucosidase from *N. crassa*. Furthermore, *N. crassa* does not have any paralogs belonging to the same clade, unlike the rest of the fungi in our study, except *Thermothelomyces thermophiles* (Fig. 2B). With only six β-glucosidases in its genome belonging to the GH3 family, *N. crassa* has thus fewer than most of the reference fungi used (Fig. 2B, Additional file 1: Fig. S1).

### Phenotypic analysis of the *gluc-1* mutation

**Fig. 3:**
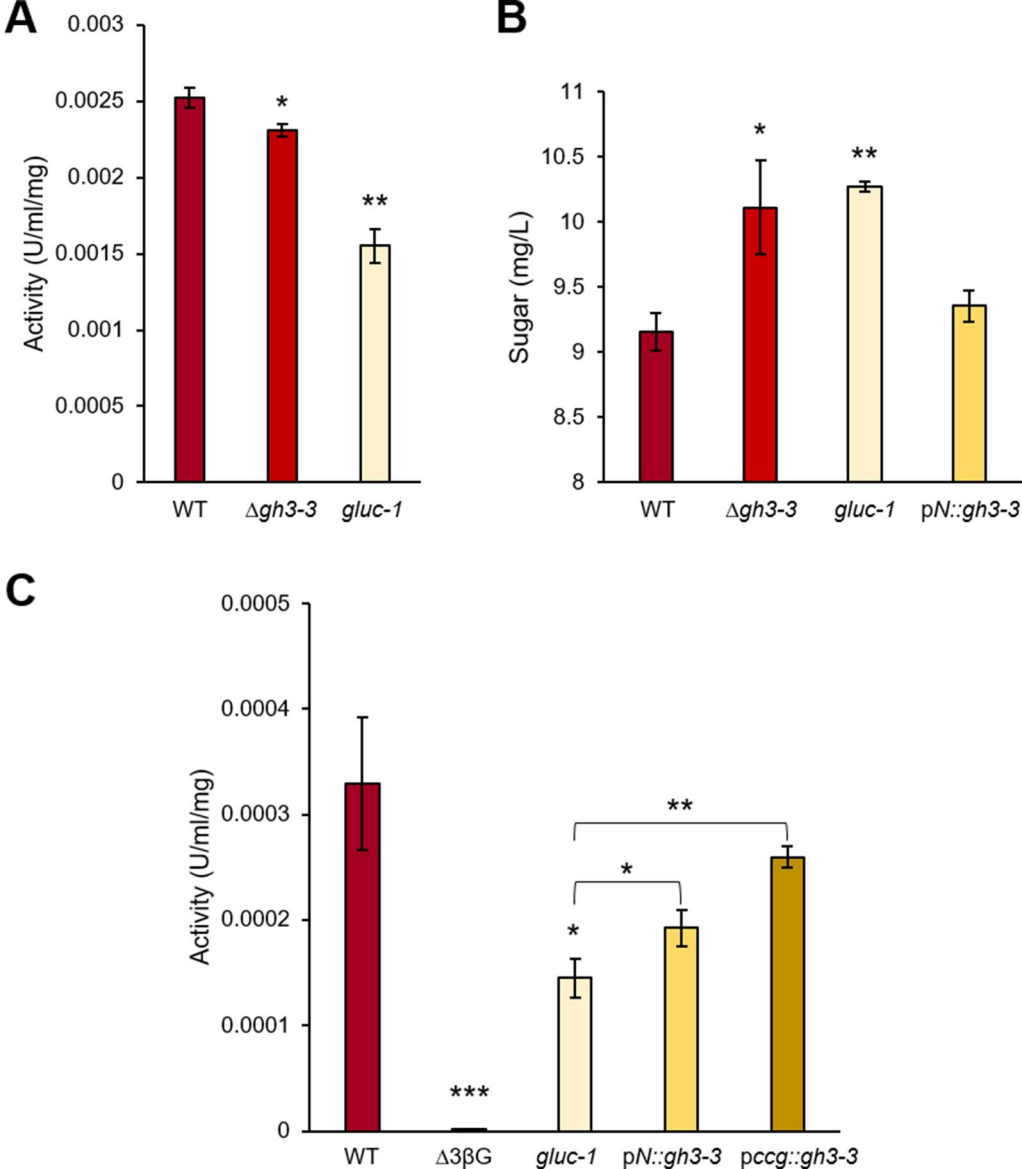
**A**. β-glucosidase activity of different *N. crassa* mutant strains after induction by Avicel cellulose. **B**. Rates of sugar utilization by different β-glucosidase mutant strains using DNS assay. Sugar concentrations in supernatant after 20 h of incubation, with a starting concentration of 10 g/L cellobiose as the sole carbon source. **C**. β-glucosidase activity of different *N. crassa* mutant strains after Avicel induction. β-glucosidase activity was determined after heat inactivation of thermolabile enzymes at 60° C for 1 min. Data are means ± standard error. Asterisks indicate statistically significant differences at p < 0.05 (*), p < 0.01 (**) or p < 0.001 (***) relative to wild-type strain, unless otherwise indicated with brackets

As a first step in our study aiming to verify whether the identified mutation is indeed causative for the overserved *gluc-1* phenotype, overall secreted β-glucosidase activity was measured in the *gluc-1* strain and compared with that of the wild-type and the ∆*gh3-3* deletion strain, which has a complete deletion of the *gh3-3* gene. As known, the β-glucosidase activity of the *gluc-1* mutant was significantly lower than that of the wild-type. Also the activity of the ∆*gh3-3* mutant was found to be significantly reduced, but not to the same extent as in the *gluc-1* strain (Fig. 3A). This observation suggests that the complete knockout of the *gh3-3* gene is similar, but does not completely copy the phenotype of the single point mutation in *gluc-1*.

### Cellobiose utilization of mutant and complementation strains

Since the ∆*gh3-3* deletion strain was shown to utilize cellobiose much more slowly than the wild-type strain (Wu et al. 2013b), we decided to analyze cellobiose utilization in the different mutants and a strain, in which the *gluc-1* mutation is complemented with the *gh3-3* gene. For this purpose, we introduced the gene *NCU08755*, encoding the β-glucosidase GH3-3, into the *gluc-1* mutant under the control of the native promoter of *gh3-3*. The rates of cellobiose utilization were analyzed in an experiment with an initial concentration of 10 g/L cellobiose, adapted from Wu et al. (2013b). Samples taken after 20 h of incubation showed that *gluc-1* and the ∆*gh3-3* deletion strain had a similarly decelerated cellobiose utilization rate, being significantly slower than the wild-type (Fig. 3B). The complemented *gluc-1* strain expressing *gh3-3* under control of the native promoter, however, showed a wild-type like rate of cellobiose utilization.

### β-glucosidase activity of mutant and complementation strains

As an essential step in this study, we next analyzed the β-glucosidase activity to ascertain whether the expression of the *gh3-3* gene was sufficient to restore the activity of the *gluc-1* strain. The ∆3βG strain, which lacks the three main β-glucosidases of *N. crassa*, was included as a control. Since the *gluc-1* mutation has been found to exclusively affect thermostable β-glucosidase activity (Eberhart et al. 1964), the β-glucosidase activity was determined after heat treatment, to eliminate interference from other, thermolabile β-glucosidases. As expected, the β-glucosidase activity in the *gluc-1* mutant was dramatically lower than that of the wild-type strain but still higher than that of the ∆3βG strain with the three main β-glucosidase genes deleted (Fig. 3C). The strain expressing *gh3-3* under control of the native promoter exhibited significantly elevated β-glucosidase activity in comparison to the *gluc-1* mutant, indicating successful complementation of the low thermostable β-glucosidase activity of the *gluc-1* strain, albeit not to the level of the wild-type strain. Considering the potential impact of expression levels on the outcomes, we decided to express *gh3-3* also under control of the strong promoter of the clock-controlled protein (*ccg-1*) (construct p*ccg::gh3-3*). When *gh3-3* was overexpressed, β-glucosidase activity was restored to wild-type levels, suggesting that the 1 kb native promoter used for native expression was not strong enough for full complementation in all conditions.

### Evaluation of different β-glucosidase mutants in both solid and liquid media

**Fig. 4:**
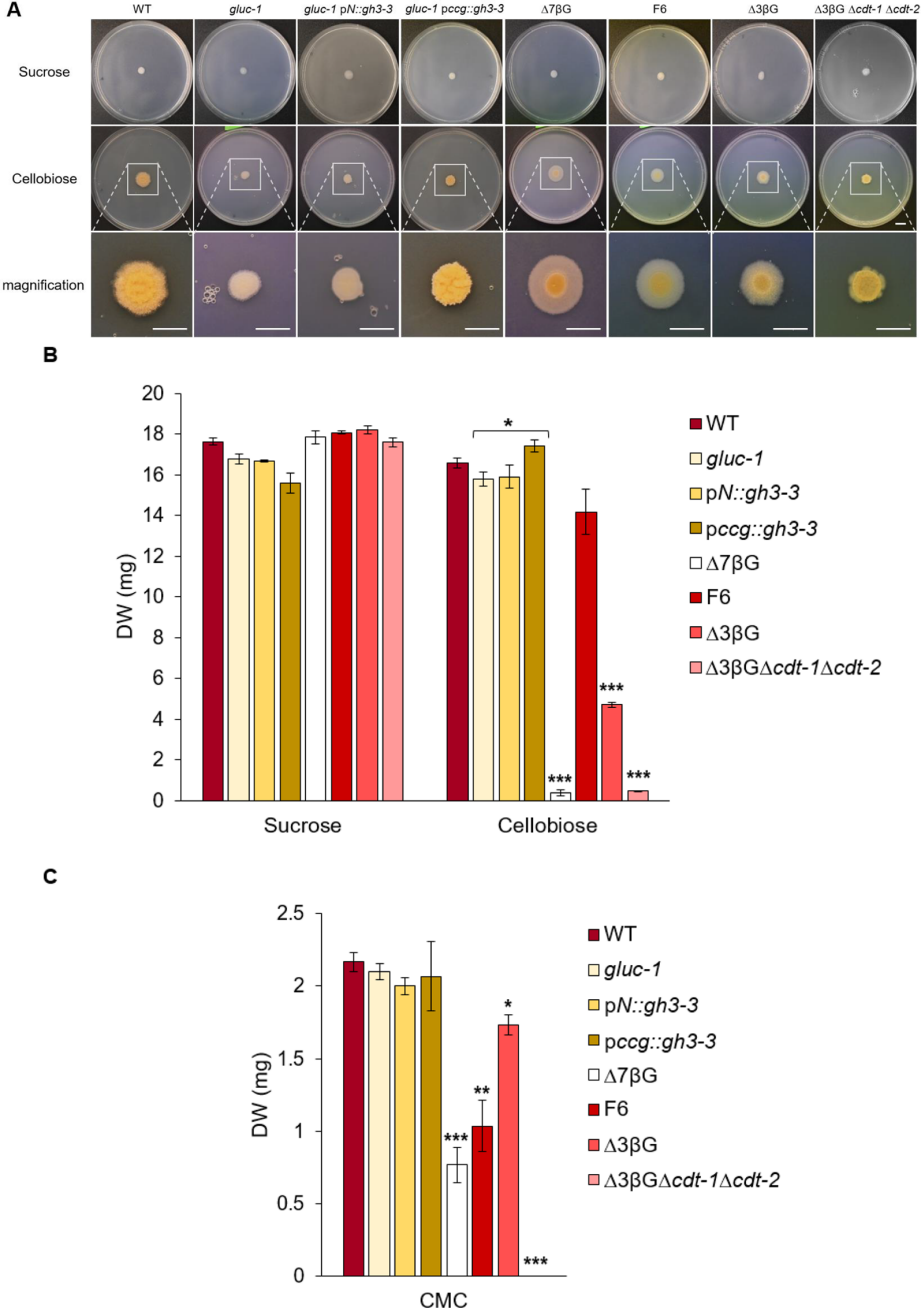
Growth assays of β-glucosidase mutant strains in plates and in liquid cultures. **A**. Growth and morphology on cellobiose plates. The same amount of conidia was spotted on VMM + 1% sugar + 0.11 M Tris (pH 8) plates and incubated at 25° C for 7 days. Tris was added to achieve colonial growth (acc. to Huberman et al. 2017). These are representative pictures from one of three independent experiments. Magnified zoom-in images of inlets are shown in the last row. Scale bars represent 10 mm. **B**. and **C**. Growth of β-glucosidase mutant strains in liquid medium. Conidia were inoculated into liquid VMM + sucrose, cellobiose (**B**) or CMC (**C**). Three biological replicates for each strain were used. Data are means ± standard error. Asterisks indicate statistically significant differences at p < 0.05 (*), p < 0.01 (**) or p < 0.001 (***) relative to wild-type strain, unless otherwise indicated with brackets

We continued our investigation by conducting a plate growth assay to measure the growth rates of various β-glucosidase mutants. Additional strains were selected in this study as a control to further understand the functioning of the cellobiose uptake and metabolism in *N. crassa* (Fig. 4A). After one week of incubation on plates using media with cellobiose as the sole carbon source, we observed an almost negligible colony growth of the *gluc-1* mutant. Slight colony development was noted in the p*N::gh3-3*-transformed strain, but more days of incubation were needed to see noticeable complementation. Only the p*ccg::gh3-3*-transformed strain exhibited a growth pattern similar to that of the wild-type strain, showing a clustered colony accompanied by conidia formation. This observation is consistent with the results of the previous β-glucosidase assay. Interestingly, this phenomenon was not observed in the deletion strains with three (∆3βG), six (F6; Wu et al. 2013a) and seven (∆7βG; Wu et al. 2013b) deleted β-glucosidase genes. These strains exhibited variable colony morphologies, with an increasingly pronounced halo effect as the number of deleted β-glucosidase genes increased. Additional deletion of the cellobiose transporter genes *cdt-1* and *cdt-2* (Galazka et al. 2010) in the ∆3βG strain background led to a very sick colony appearance, which confirms that these two major *N. crassa* cellobiose transporters CDT-1 and CDT-2 do indeed play a very important role in cellobiose utilization.

Surprisingly, the *gluc-1* mutant showed only a very mild growth phenotype in liquid media containing cellobiose as sole C-source (Fig. 4B), similar also to the F6 mutant, carrying only the functional *gh3-3* gene. While no improvement was observed with the complementation construct expressed under control of the native promoter, the p*ccg*::*gh3-3* strain showed again an elevated level of growth. In contrast, the ∆3βG strain showed a strongly inhibited growth pattern, which was further reduced by additional deletion of the two cellodextrin transporters (∆3βG∆*cdt-1*∆*cdt-*2) to extremely low levels similar to the full β-glucosidase knockout strain ∆7βG*.* Overall, these observations reveal that a minimal amount of β-glucosidase activity is needed to sustain robust growth in liquid cellobiose media, indicating that the redundant system of *N. crassa* is quite resilient towards loss-of-function of even several enzymes as long as the uptake system is functional.

We also tested the same mutants in liquid media containing 1% cellulose (CMC) for 8 days (Fig. 4C). In general, all strains, including the wild-type, exhibited much lower biomass accumulation when compared to growth on cellobiose. In this condition, there was no significant difference in biomass accumulation between the wild-type, *gluc-1*, p*N::gh3-3*, and p*ccg::gh3-3* strains. Interestingly, growth on CMC was notably reduced in the F6 strain, which contrasted with its performance on cellobiose. While growth was again severely hampered in the ∆7βG strain, almost complete loss of growth was observed only for the strain missing the cellodextrin transporters (∆3βG∆*cdt-1*∆*cdt-*2), further corroborating their importance in the uptake of slowly released cellobiose. Overall, these data imply that the strains can optimize their adaptation to diverse environments through ongoing internal regulatory processes of cellobiose metabolization, while cellobiose uptake is an essential step.

### Cellulase activity of the β-glucosidase mutant strains

**Fig. 5:**
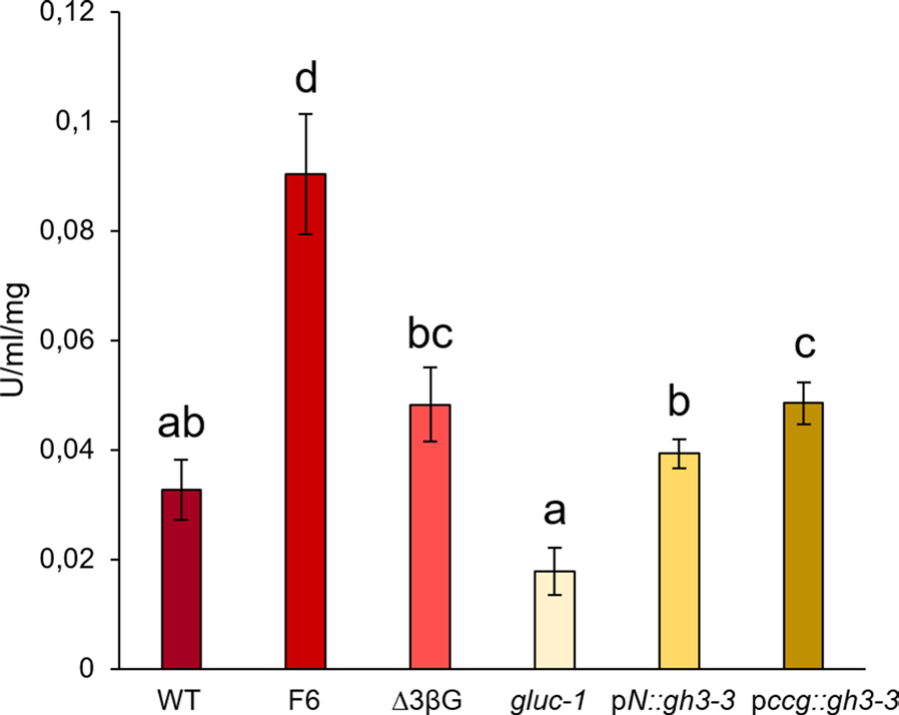
Cellulase activity of different *N. crassa* mutant strains after Avicel induction. Cellulase activity was determined using AZO-CM-Cellulose reagent. Data are means ± standard error. Different letters indicate statistically significant differences

We next measured the cellulase activities in the different mutants. A previous study had confirmed that cellulose degradation products can induce cellulase gene expression in *N. crassa* with an even higher induction observed in the ∆3βG mutant, which was attributed to the accumulation of inducer molecules within the mutant cells (Znameroski et al. 2012). However, this phenotype had not been observed in the *gluc-1* mutant (Eberhart et al. 1964). In our assays, cellulase activity of the F6 strain was found to be much higher than in the wild-type (Fig. 5), even higher than of the ∆3βG mutant, which also showed elevated cellulase activity, confirming the results by Znameroski et al. (2012). Activity of the *gluc-1* mutant, however, was slightly reduced compared to the wild-type, which was complemented again by both *gh3-3* misexpression strains. Interestingly, cellulase activity of the p*ccg::gh3-3* strain even slightly exceeded wild-type levels, although not as much as the F6 mutant.

## Discussion

By the action of endoglucanases and exoglucanases on cellulose, the main component of the cell walls of plants, this insoluble complex sugar is broken down into soluble cellodextrins, including cellobiose. Subsequently, β-glucosidases take over to hydrolyze these into glucose monomers. In this study, we analyzed the classical *gluc-1* mutant of *N. crassa*, selected for having greatly reduced thermostable β-glucosidase activity, and compared its phenotype with that of other major β-glucosidase mutants.

The *gluc-1* mutation was putatively located in the *gh3-3* gene that encodes GH3-3/BGL6, one of the main extracellular β-glucosidases in *N. crassa* (McCluskey and Baker 2022). Therefore, our starting hypothesis was that this mutation is causative for the *gluc-1* phenotype. Lowered β-glucosidase activity and cellobiose utilization in the ∆*gh3-3* mutant support this hypothesis. However, although the phenotype of the ∆*gh3-3* deletion strain was similar to the *gluc-1* mutant, it was not able to phenocopy it entirely, suggesting that the *gluc-1* mutation affects protein function differently than a complete deletion of the gene. This indicates for additional and dominant, but so far unknown, effects caused by the point mutation. Potentially, the mutated β-glucosidase may influence extracellular enzyme secretion, as was observed in a previous study (Ribeiro et al. 2013). In the ∆*gh3-3* deletion strain, on the other hand, a genetic compensatory response could be occurring through activation of the expression of alternative β-glucosidases, as observed with the deletion of the β-glucosidase gene *bglC* in the plant pathogen *Streptomyces scabies* (Deflandre et al. 2020). Moreover, the deletion of the β-glucosidase gene *cel3g* in *T. reesei* was found to enhance the expression of the β-glucosidase genes *cel1a* and *cel1b* (Zou et al. 2018). In *N. crassa*, this compensatory response was already observed with expression of the β-glucosidase *gh3-6* gene (*NCU07487*) being increased fourfold in the ∆3βG triple mutant, supporting this hypothesis (Znameroski et al. 2012). Alternatively, it cannot be ruled out that the phenotypic differences observed between ∆*gh3-3* and *gluc-1* might be due to the presence of secondary mutations that were created either during the UV mutagenesis leading to the *gluc-1* strain or during the knock-out of *gh3-3* in the ∆*gh3-3* strain, which has been previously observed in *N. crassa* (Montenegro-Montero et al. 2023).

For the above reasons, we decided to focus on complementation assays with the *gh3-3* gene instead. Despite the fact that the expression strength of the construct under control of the 1 kb native promoter might have been lower than necessary, it complemented the deficiency of the *gluc-1* phenotype partly to fully in terms of β-glucosidase and sugar utilization activities. The use of the stronger p*ccg* promoter improved the results in some of the experiments. Plate growth assays revealed that only the *gluc-1* strain overexpressing *gh3-3* showed growth comparable to the wild-type strain on cellobiose. Furthermore, complementation of growth in liquid medium with cellobiose as the sole carbon source was only achieved when *gh3-3* was overexpressed and Avicel-induced thermolabile β-glucosidase activity also showed a similar trend. Overall, these experiments strengthened our confidence that *gh3-3* is the causative gene for the *gluc-1* phenotype after all.

Intriguingly, overexpression of *gh3-3* in the *gluc-1* mutant strain led to a significant increase in cellulase activity compared to the wild-type strain, which did not occur in either the *gluc-1* strain or the strain expressing *gh3-3* under the native promoter. A similar observation was made in *Trichoderma reesei* when heterologously expressing a β-glucosidase gene from *Penicillium decumbens* (Ma et al. 2011). In addition, increased hydrolysis efficiency of the enzyme system on filter paper was accomplished by overexpressing β-glucosidases in the genus *Penicillium* (Yao et al. 2016). These data support the idea that mis-expression of β-glucosidases often lead to regulatory effects, probably since they directly affect the quantity of available inducer molecules. Also the *gluc-1* strain was interpreted to have a regulatory defect, with the *gluc-1* gene being dominant in expression over the wild-type *gluc-1*^+^ allele (Eberhart et al. 1964), indicating that even the rather small change by a single amino acid substitution could drastically alter the activity of the enzyme.

**Fig. 6:**
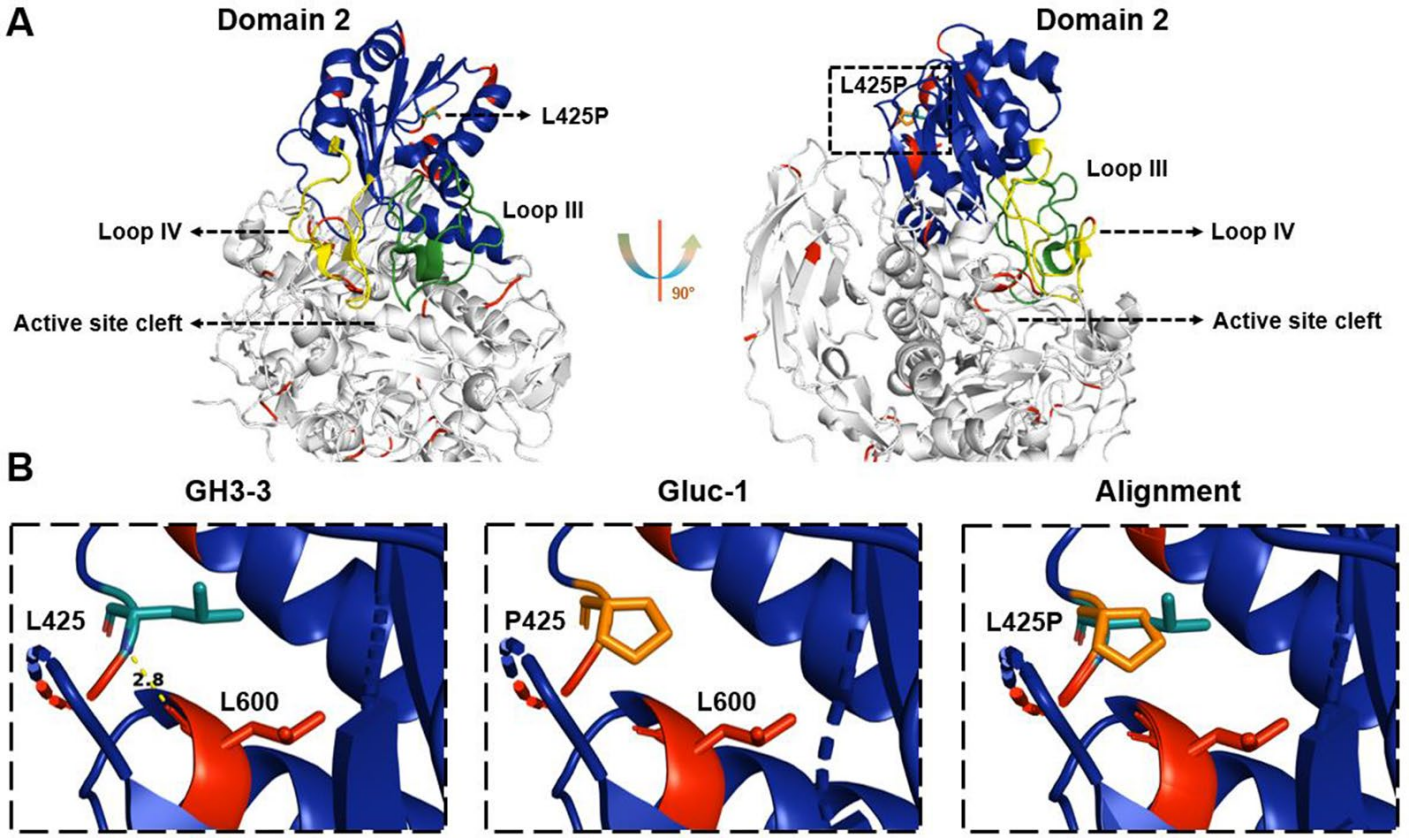
Overview comparison of domain 2 between the GH3-3 crystal structure and the Gluc-1 model.** A**. Domain 2 in GH3-3 and Gluc-1 alignment. The Gluc-1 model calculated from SWISS-MODEL (Waterhouse et al. 2018) was aligned to GH3-3 crystal structure (5nbs.1.A) (Karkehabadi et al. 2018). The α/β region of domain 2 is colored in blue. Loops III and IV are colored in green and yellow, respectively. Red color indicates mismatched positions between the two structures. The L425P point mutation is highlighted in orange (black box). **B**. Mutated position in the alignment between GH3-3 and Gluc-1. The structure in the L425P region in the structural alignment between GH3-3 and Gluc-1 was compared. The hydrogen bond between L425 and L600 in GH3-3 is colored in yellow within 2.8 Å

The *gluc-1* mutation is located in the second domain of the GH3-3 structure, which also includes the two important loops III and IV. Together, both loops constitute one side of the active site cleft (Fig. 6; Additional file 2: File S1; Karkehabadi et al. 2018). The *gluc-1* mutation is located on the opposite side, but due its position in the highly ordered backbone of domain 2, it might be critical for positioning the two loops, which are important for catalytic activity. This fact suggests that it could indeed be a crucial residue for protein function. The mutated enzyme may for example block cellobiose by unproductive binding, but further experiments are needed to test this hypothesis.

The fact that the sole presence of the *gh3-3* gene in the sextuple mutant F6 strain was sufficient to grow similarly as the wild-type strain in liquid medium with cellobiose suggested that the β-glucosidase GH3-3 is the main extracellular β-glucosidase in *N. crassa*, as observed by Wu et al. (2013b) and that its presence is sufficient for the utilization of cellobiose (at high concentrations) from the environment. The lack of wild-type-like growth on cellobiose plates of this sextuple mutant can potentially be explained by the fact that effective cellobiose concentrations around the hyphae drop substantially over time, likely below the affinity thresholds of GH3-3, which would then not be able anymore to convert it efficiently to glucose. Similarly, the observed reduced growth of the F6 strain on cellulose could be because also in this condition the effective concentrations of cellobiose are likely well below the affinity thresholds of GH3-3. The higher cellulase activity in this mutant compared to the wild-type could then be due to the presence of more intact cellobiose being taken up and acting as an inducer (similar to what has been stated by Znameroski et al. 2012).

After the interesting observation of a larger halo on cellobiose plates with an increasing number of β-glucosidase gene deletions (thus lower number of functional intra- and extracellular β-glucosidases), we hypothesized that this loss of overall activity could trigger radial extension of the mycelium in search for new carbon sources to survive, being more urgent when less β-glucosidases are present. A similar adaptive morphology at the colonial level was found in *Microcystis aeruginosa* for the adaptation to nutrient availability (Yan et al. 2021; Feng et al. 2022), and nutrient stress may act as an inducer to switch to exploratory hyphae, as has been found in the case of the plant pathogenic fungus *Zymoseptoria tritici* (Francisco et al. 2019). Our phylogenetic analysis of GH3 β-glucosidases showed that the family in *N. crassa* is one of the smallest (6) of the reference fungi used, even compared to that of the well-characterized set of β-glucosidases from *T. reesei* (9) (Pang et al. 2021). Moreover, it has at most one protein representing each cluster of the phylogenetic tree, indicating that there is little redundancy of β-glucosidases in this organism, which is typical for *N. crassa* due to highly active RIP mutagenesis of gene duplications (Galagan and Selker 2004) and could explain the phenotypic effect of halo size between mutants differing in a single deleted gene.

As key players in cellulose and cellobiose assimilation, the cellodextrin transporters CDT-1 and CDT-2 have been extensively studied (Galazka et al. 2010; Cai et al. 2014; Znameroski et al. 2014; Lin et al. 2017). Deletion of the genes encoding the two major cellodextrin transporters *cdt-1* and *cdt-2* of *N. crassa* in the ∆3βG strain led to a very sick phenotype of the colony, probably due to the total inability to acquire the carbon source from the medium, neither as glucose after extracellular β-glucosidase activity, nor as cellobiose via uptake over the plasma membrane. In addition, growth in liquid medium of this strain with either cellobiose or cellulose as the sole carbon source was similar or even inferior to that of the ∆7βG strain lacking all β-glucosidase genes, supporting that both cellodextrin transporters play a major role for the uptake of cellobiose from the surroundings.

In conclusion, we demonstrated in this work that the phenotype of the classical *gluc-1* mutant mutation is due to a mutation located in loop III of the *gh3-3* gene and is complemented only when the wild-type version of the gene is strongly expressed. GH3-3 is one of six *N. crassa* β-glucosidases belonging to the GH3 family, with GH3-4, another major secreted enzyme, being its closest homolog in *N. crassa.* Overall, our study confirmed that GH3-3 is a major extracellular β-glucosidase in *N. crassa*, being both necessary and sufficient for extracellular cellobiose utilization. Moreover, we observed for the first time that the colony phenotype of multi-β-glucosidase mutants depends on the number of deleted BGL genes and show that the two main cellodextrin transporters CDT-1 and CDT-2 are essential for cellobiose utilization when the three major β-glucosidases of *N. crassa*—the intracellular protein GH1-1 and the two extracellular proteins GH3-3 and GH3-4—are absent. These findings expand the understanding of the molecular mechanisms of cellulose utilization and connect enzymatic and transporter players, which is essential for the reuse of agricultural residues and will be beneficial both from an economic and environmental point of view.

### Supplementary Information

**Additional file 1: Fig. S1. **Phylogenetic analysis of GH3 family protein with β-glucosidases and β-xylosidases from various filamentous fungi. The unrooted neighbor-joining tree was created with MEGA11. NCBI accession numbers are indicated. *N. crassa *GH3-3 is shown in brown bold. The other *N. crassa *β-glucosidases are shown in bold. GH3-7 and GH3-8 (grey bold) are β-xylosidases from *N. crassa*, and clustered with β-xylosidases from other fungi. The β-glucosidase GH1-1 (of the GH1 family) was used as an outgroup. Bootstrap values above 70 and supporting a node used to define a cluster are indicated.

**Additional file 2.** File S1. Alignment of the predicted Alphafold model of the Gluc-1 protein with the crystal structure of GH3-3.

## Data Availability

All data generated or analyzed during this study are included in this published article [and its supplementary information files].
