# Supplementary material for: Unveiling a classical mutant in the context of the GH3 β-glucosidase family in Neurospora crassa
Source: AMB Express. 2024 Jan 5;14:4. doi: 10.1186/s13568-023-01658-0 (PMC10770018; doi:10.1186/s13568-023-01658-0)
Supplement: Supplementary file 1 — Additional file 1: Fig. S1. Phylogenetic analysis of GH3 family protein with β-glucosidases and β-xylosidases from various filamentous fungi. The unrooted neighbor-joining tree was created with MEGA11. NCBI accession numbers are indicated. N. crassa GH3-3 is shown in brown bold. The other N. crassa β-glucosidases are shown in bold. GH3-7 and GH3-8 (grey bold) are β-xylosidases from N. crassa, and clustered with β-xylosidases from other fungi. The β-glucosidase GH1-1 (of the GH1 family) was used as an outgroup. Bootstrap values above 70 and supporting a node used to define a cluster are indicated. [file 13568_2023_1658_MOESM1_ESM.pdf]

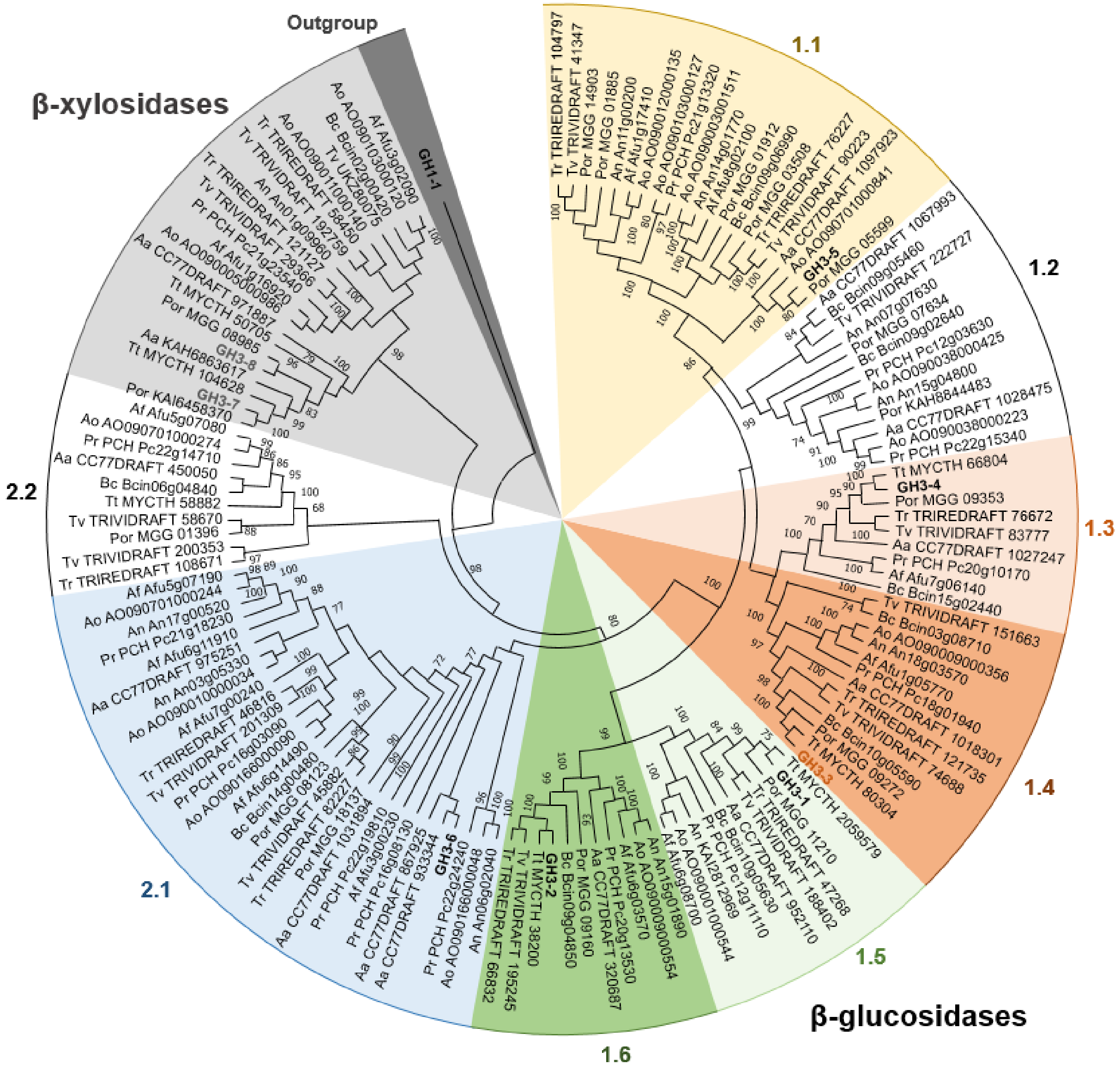

Fig. S1. Phylogenetic analysis of GH3 family protein with β-glucosidases and β-xylosidases from various filamentous fungi. The unrooted neighbor-joining tree was created with MEGA11. NCBI accession numbers are indicated. *N. crassa* GH3-3 is shown in brown bold. The other *N. crassa* β-glucosidases are shown in bold. GH3-7 and GH3-8 (grey bold) are β-xylosidases from *N. crassa,* and clustered with β-xylosidases from other fungi. The β-glucosidase GH1-1 (of the GH1 family) was used as an outgroup. Bootstrap values above 70 and supporting a node used to define a cluster are indicated.
